# Dual ICA to extract interacting sets of genes and conditions from transcriptomic data

**DOI:** 10.1145/3584371.3612968

**Published:** 2023-10-04

**Authors:** Sanjeevani Choudhery, Thomas R. Ioerger

**Affiliations:** Department of Computer Science and Computer Engineering, Texas A&M University, College Station, TX, 77840

**Keywords:** RNA-Seq studies, Independent Component Analysis (ICA), Co-expression analysis, Gene-condition sets

## Abstract

One of the challenges in RNA-Seq studies is finding subsets of genes that share a common mechanism of action or are associated with a regulon/pathway. Existing approaches often extract modules that reflect quantitative similarities (such as genes with correlated log-fold-changes) but do not adequately capture biological significance. In this work, we propose the Dual ICA methodology, which provides an *agnostic* way to extract “interacting modules” composed of sets of genes and conditions that exhibit strong associations. Dual ICA involves performing Independent Component Analysis (ICA) twice, once on the genes and once on the conditions. Using the resulting signal matrices, we extract respective sets of genes and conditions. The interaction between these sets is quantified using the coefficients from a linear regression and significance is determined through the Wald test and Z-score filtering. These coefficients are equivalent to the outer product of independent components obtained from the two signal matrices. Not only do the gene sets extracted align with known regulons, but the significant interacting modules they instantiate also encompass conditions that influence the expression of these regulons through shared mechanisms of action. Compared to traditional unsupervised clustering methods, Dual ICA demonstrates superior performance and provides explicit gene-condition sets for exploring functional relationships.

## INTRODUCTION

Transcriptional profiling enables a high-throughput readout of behavior or phenotype, allowing one to cluster genes based on their similar responses to different environmental conditions, stresses, drug treatments, etc. These empirical gene sets provide insights into the biological roles of genes through pathway membership and can even reveal new pathway associations. Integration of other omics datatypes such as metabolomic [1, 2], and proteomic, and TnSeq essentiality data [3] with RNASeq may enhance our understanding of functional relationships among gene sets. Different gene sets often exhibit a signal [4] (e.g. dysregulation, or conditional essentiality) associated with specific conditions or treatments (phenotypes). By analyzing response patterns, we can not only identify associations or interactions between genes but also cluster the conditions themselves. In some cases, we expect certain treatments to produce similar responses, while in other cases, we want to automatically associate new treatments (such as profiling transcriptional responses to new inhibitors from high-throughput screening) with existing datasets to determine their similarity. Clustering conditions can provide additional insights into the mechanisms of action and their effects on pathways. However, similarity among conditions is not uniformly represented across all genes, as it is multidimensional.

Thus, the challenge is to find these signals and extract “interacting modules” where a subset of genes behave similarly in a subset of conditions and reflect the common mechanism or pathway affected by the clustered conditions. This is the goal of various biclustering methodologies [5]. However, these relationships are difficult to find because relevant determinants of similarity among conditions cannot be determined without knowing relevant gene sets a priori and vice versa. In this paper, we will show how these interacting modules can be extracted by applying Independent Component Analysis (ICA) to find clusters of genes and conditions, and test these to extract significant interacting modules.

### 1.1 Previous Work

Various methods, such as WGCNA [6], have been used to cluster genes in an unsupervised manner based on co-expression similarity and hierarchical clustering. These methods have been successfully applied in different biological contexts, including cancer, mouse genetics, yeast genetics, and brain imaging data analysis. However, these methods produce gene clusters without much insight into the associated conditions that lead to their dysregulation.

Principal Component Analysis (PCA) [7] is a commonly used technique to rotate data, presenting new axes as linear combinations of the original axes. Clustering is typically performed using the extracted principal components (PCs). However, PCA does not offer a decomposition to identify relationships among conditions as well as UMAP. Uniform Manifold Approximation and Projection (UMAP), also a dimensionality reduction technique, captures the structure of high-dimensional data and creates a low-dimensional representation while preserving relationships [8]. It effectively clusters single-gene mutant libraries based on cellular activities, pathways, protein complexes, and protein-protein interactions [9]. However, UMAP's primary focus is on clustering conditions, and it does not provide detailed insights into changes in the behavior of subsets of genes across different conditions.

A popular approach to discover interacting modules of genes and conditions is biclustering [10]. In contrast to the methods above, it simultaneously groups rows and columns of a data matrix. Various approaches have been proposed to do this in gene expression analysis. Direct Methods such as the Iterative Signature Algorithm (ISA) [11] and the Bimax algorithm iteratively identify optimal submatrices [12]. Probabilistic methods such the BicMix algorithm use Bayesian frameworks or probabilistic models to capture patterns [13]. Matrix Factorization Methods such as the Non-negative Matrix Factorization (NMF), decompose the input matrix to reveal biclustering patterns [13]. Notable differences among these methods are whether they enforce a disjoint clustering or allow overlaps and whether they require all members to be clustered. In our experimentation with biclustering, we observe that it is susceptible to noise as condition clusters often fail to capture mechanisms of action, and gene clusters may not align with known regulons/pathways.

Independent Component Analysis (ICA) [14], is another data decomposition technique that aims to find independent non-Gaussian signals in a data matrix X. It decomposes X into a signal matrix S and a mixing matrix A, such that X = S x A. Unlike PCA, ICA optimizes for non-normality (non-Gaussianity) of each axis (or minimization of mutual information [15]). The mixing matrix A contains coefficients that determine the weights used in the linear combination of source signals in S. This allows for the extraction of better-defined signals in the data than PCA and the other methods defined above [16] .

Frequently, clusters extracted in the resulting IC space are more accurate than UMAP and biclustering and encompass conditions that function through a similar mechanism of action. It can also be used to extract gene sets that coincide with known regulons. Calhoun [17] performed basic ICA with fMRI data to make group inferences implemented in the GIFT software. Furthermore, Liu [18] incorporated fMRI data with full SNP arrays and used ICA on this multimodal data to find a specific factors to study further.

Sastry [19] employed ICA in a semi-supervised manner to extract gene sets (iModulons) from an *E. coli* transcriptomic dataset for various growth conditions. These iModulons aligned with known transcriptional regulators. Genes were associated with iModulons using the D’Agostino test for normality and outlier removal until a predetermined K^2^ cutoff was reached. The authors validated the iModulons using the mixing matrix A, to determine "activities" of iModulons on individual conditions. However, they did not cluster the experimental conditions themselves, relying on prior knowledge and assumptions about condition similarity.

While this semi-supervised approach works well when treatment similarities are known, analyzing large 'omics datasets with unknown treatment similarities, such as transcriptional responses to new drugs or gene knockouts, requires an agnostic determination of the relationships among individual conditions.

### 1.2 Dual ICA Methodology

In this work, we propose the Dual ICA methodology as a robust and innovative approach for extracting interacting modules of genes and conditions. Our method incorporates two ICA decompositions, enabling us to determine associations between independent components extracted from genes (gene ICs) and condition ICs, thereby providing a more comprehensive understanding of the relationships between genes and conditions.

A similar method was used by Gupta et al., [20] for biclustering of fMRI data, where the authors use ICA to generate row-column clusters, but they use only one ICA.

The effectiveness of ICA comes from its ability to extract specific gene signals across conditions. Suppose there exists a specific group of genes that exhibit strong and coordinated upregulation or downregulation in a set of conditions, these genes would manifest as a strong "signal" within one of the ICs obtained through ICA analysis on those conditions. This phenomenon relies on appropriately rotating the axes (resulting ICs) as a linear combination of the conditions where these gene groups are involved or projecting them onto the precise linear combination of the conditions. Hypothetically, these genes would appear as "outliers" within this IC, characterized by log fold changes (LFCs) that significantly deviate from the central mode observed in the bell curve distribution of expression values for the remaining genes in the genome, within this new IC space. The same principle applies when conducting ICA on genes. By identifying the ideal linear combination of the implicated genes, conditions exhibiting strong signals within a subset of genes would manifest as outliers in the LFC distribution within the gene ICA-derived space.

We enhance the standard ICA methodology used in previous studies, by utilizing the matrix outer product to identify “interacting modules” of *interacting* genes and conditions. The outer product in our approach is equivalent to the coefficients obtained from a linear regression of the log fold changes (LFCs) on membership in these interacting modules. Significant coefficients, determined by the Wald test [21] and Z-score filtering, extract compelling associations between of groups of conditions with similar mechanism of action and the gene sets or regulons associated with the mechanism.

We applied this methodology to transcriptomic datasets from two different species, *Escherichia coli* and *M. tuberculosis*, including transcriptional responses to drug treatments [2]. In both cases, the resulting interacting modules effectively captured conditions with a shared mechanism of action, along with relevant associated genes. Notably, despite the diverse origins of the samples in these datasets, the identified modules showed strong alignment with known pathways and regulons, surpassing the performance of traditional unsupervised clustering methodologies.

Given that the Dual ICA method identifies drug classes and their associated genes consistent with known associated pathways, we can infer some accuracy for drug classes without known pathways. Therefore, this method allows us to *make unbiased inferences about* *genes playing a role in certain treatments / stress response pathways while also uncovering commonalities between the treatments themselves*. By accurately capturing associations between drug classes, their targets, and known associated pathways, our approach provides valuable insights for drug discovery and mechanism exploration.

## 2 METHODS

### 2.1 Independent Component Analyses

#### 2.1.1 Preprocessing

The input to the Dual ICA methodology is a transcriptomic data matrix M of size n x m, where n is the number of genes and m is the number of conditions or treatments. The data consists of log-fold-changes (LFCs) of each gene in each condition (usually with multiple replicates), as estimated by tools like DeSeq2 [22] or Limma [23], computed relative to the reference condition in the experiment.

This data matrix is normalized (standardized) per column, i.e., per condition. Then we set any LFC greater than 6, to 6 and any LFC less −6 to −6, which limits the impact of large outliers. The cutoff magnitude of 6 was determined heuristically through a comparison of multiple datasets. Most of the datasets showed LFC values ranging between −6 and 6. Among the 260 conditions in the PRECISE database, only 7 datasets displayed around 20 outlier values (with a magnitude > 6), primarily associated with a single study. However, the remaining datasets had an average of approximately 1 outlier each. These outliers, falling outside the specified range, biased the decompositions and were unlikely to represent biologically realistic data.

#### 2.1.2 Decomposition of Data Matrix

Let the result of an ICA defined using an r number of components on any data matrix X be a decomposition

$$X_{\left[nXm\right]}=S_{\left[nXr\right]}\;A_{\left[rXm\right]}$$

where S is a ‘signal matrix’ that reflects the r signals present in the n genes across the conditions, and A is a ‘mixing matrix’ that shows the mixture of m conditions that make up the r signals [24].

We perform ICA on our data matrix two times. Once with data matrix M using k condition-based independent components (condition ICs):

$$M_{\left[nXm\right]}=G_{\left[nXk\right]}\;A_{\left[kXm\right]}$$

and once with the transpose of the data matrix, using 1 gene-based independent components (gene ICs):

$$M_{\;\;[mXn]}^{T}=C_{\left[mXl\right]}\;B_{\left[lXn\right]}$$

The result is two signal matrices, one which will allow for a grouping of genes and one that will allow for a grouping of conditions. In ICA, the underlying assumption is that the sources are statistically independent and non-Gaussian. The measure of Gaussianity used is the omnibus K^2^ value from the D’Agostino test for kurtosis and skewness [25]. The D’Agostino test was used over the Shapiro-Wilk test, the traditional test of normality, because the very high power of Shapiro Wilk [26] made it excessively sensitive to outliers. The D'Agostino test provided the appropriate sensitivity due to its consideration of higher moments in the data distribution. Higher K^2^ values indicate greater non-Gaussianity, suggesting that the independent component tested captures a distinct signal. Thus, the K^2^ value for each independent component produced by ICA was calculated and examined as the number of independent components increased. This was done twice, once on signal matrix G and once on signal matrix C. The point at which the rate of increase diminished, determined using the kneedle algorithm [27], was used as the number of components (k and l) in the respective decompositions.

### 2.2 Quantifying IC Associations

#### 2.2.1 Outer Product Matrix Calculation

The next step is to associate the k condition ICs (columns of signal matrix G) with the l gene ICs (columns of signal matrix C) to determine which clusters of genes associate with which clusters of conditions (i.e. interacting modules). The association of a given condition IC $G_{i}$ (of size number of genes[g]x1) with gene IC $C_{j}$ (of size number of conditions [c]x1) is represented by the calculation:

(1) $$assoc(i,j)=\sum_{a,b}(G_{i}\;⊗\;C_{j})⊙M$$

The outer product (⊗) of the two selected vectors ($G_{i}$ and $C_{j}$) is computed and pointwise multiplied (⊙) with the LFC matrix. All elements a,b of the resulting matrix are summed into one value expressing the association between the two ICs. The result of the association calculation for every pair of condition and gene IC results in a matrix of size kxl, where each value represents the association of a given condition IC $G_{i}$ and gene IC $C_{j}$. Multiplying the data matrix by the result of the outer product puts highest weight on the LFCs that are the either both the highest or both the lowest in both gene set *i* and condition group j. We then sum all the LFCs in the data matrix, which effectively reflects the strength of the signal in those entries in the matrix.

#### 2.2.1 Coefficients from Linear Regression

The association calculation is equivalent to a regression where the target values are obtained by melting the input matrix to get LFCs for each gene-condition combination and covariates are the product of the k mappings of gene g in G and l mappings of condition c in C:

(2) $${LFC}_{g,c}=\beta_{0}+\sum_{i=0}^{k}\sum_{j=0}^{l}\vartheta_{\mathrm{ij}}G{\left[i\right]}_{g}{C[j]}_{c}$$

The solution to any $Y=\beta^{T}X$ is $\beta =\frac{X^{T}Y}{X^{T}X}$, i.e., the estimate for $\beta_{k}$ is $Cov(x_{k},y)$ minus some miscellaneous covariance terms divided by a normalizing constant from the inversion of the covariance matrix. The miscellaneous covariances in this model are 0, i.e., there is no collinearity between the variables in our linear model. Therefore, any given coefficient $\beta_{k}$ would follow the equation

$$\beta_{k}\propto \sum_{i=0}^{n}(x_{k_{i}}-\bar{x_{k}})y_{i}$$

Following this,

$$\vartheta_{ij}\propto \sum_{z=0}^{g*c}({G[i]}_{z}\cdot {C[j]}_{z}-\bar{G[ı]\cdot C[j]})\cdot {LFC}_{z}.$$

**Figure 1:**
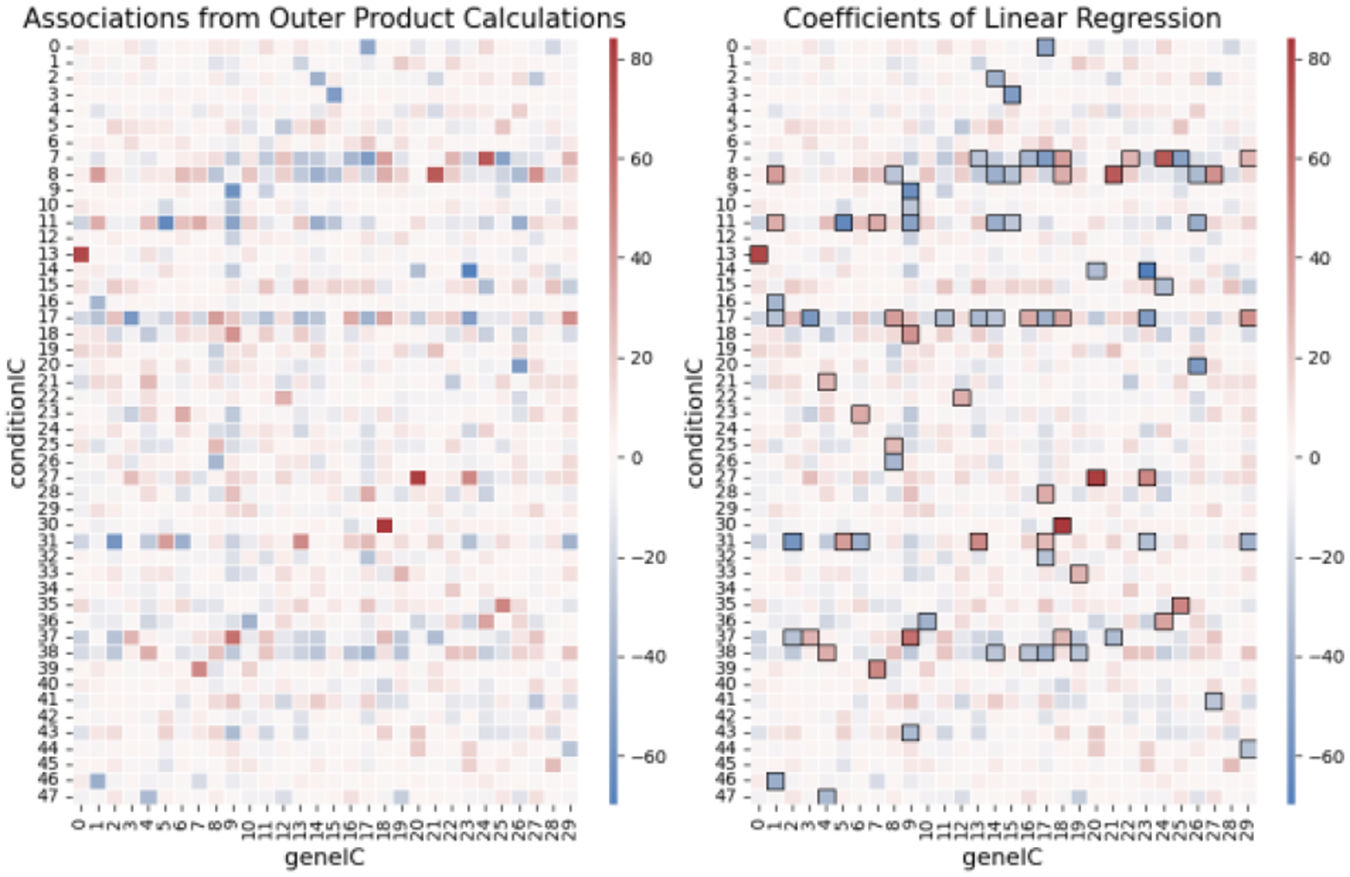
Heatmaps of the associations of geneICs and conditionICs quantified using the outer product method (left) and as coefficients of the linear regression (right) on the PRECISE dataset. The associations outlined in black are those found to be significant.

The input matrix is centered automatically before the ICA is performed. Thus, μ=0 for any component in an ICA decomposition and any G[i] and C[j] do not correlate, thus simplifying the previous equation to

$$\vartheta_{ij}\propto \sum_{z=0}^{g*c}{G[i]}_{z}\cdot {C[j]}_{z}\cdot {LFC}_{z},$$

which is the outer product calculation for any point (i,j) in the association matrix. Similarly, to the outer product calculation, the resulting coefficients of the fitted linear regression can restructured as matrix of size kxl. Figure 1 shows the matrices of the outer product calculation and coefficients from a fitted linear regression (combinations of independent components extracted from transcriptional dataset from *E. coli*, vida infra). They are equivalent.

#### 2.2.2 Statistical Significance

If there are k condition clusters and l gene clusters, then there can be as many as kxl interacting modules to test, only a subset of which should be significant. The linear regression not only provides association information as the outer product does, but it also allows us to quantify significance of every association. These coefficients reflect levels of gene cluster activity in various sets of conditions. We obtain p-values from a Wald test to determine coefficients (interactions) are significantly different from 0, and then apply the Benjamini-Hochberg correction for multiple testing [28]. Although coefficients may be significantly different than 0, they may not be large enough to truly reflect biological significance. Therefore, analogous to filtering performed in RNA-Seq studies, we apply a filter of a ∣Z-score∣ >= 2. In Figure 1, the black boxes reflect these significant associations.

### 2.3 Extracting Interacting Modules

To identify which genes are associated with each condition IC (i.e. clustering genes by which condition(s) they are most strongly dysregulated in), we analyzed the distribution of coefficients in the signal matrix G and looked for outliers (i.e. in tails of a bell curve). For every independent component $G_{i}$ in the k condition ICs, the omnibus K^2^ value is calculated using the D’Agostino test for kurtosis and skewness [29]. The K^2^ values reflect a combination of skewness and kurtosis and are assumed to follow a Chi squared distribution which is used to calculate the significance (p-value) of the K^2^ value. For a given component, two associated gene clusters were extracted by repetitively removing genes with the highest absolute coefficient (which are effectively outliers) and adding them to a positive set or negative set based on the sign, until the remainder of the coefficients followed a normal distribution per the D’Agostino test (i.e. until the p-value>=0.05). For terminology, we indicate the sub-clusters with signed subscripts in the labels. For example, G_8+_ represents the cluster of genes with positive coefficients associated with condition IC 8. This procedure identifies elements associated with an IC as an outlier with extreme values in the tails of the distribution and removes them progressively until the remaining values are approximately normally distributed.

**Figure 2:**
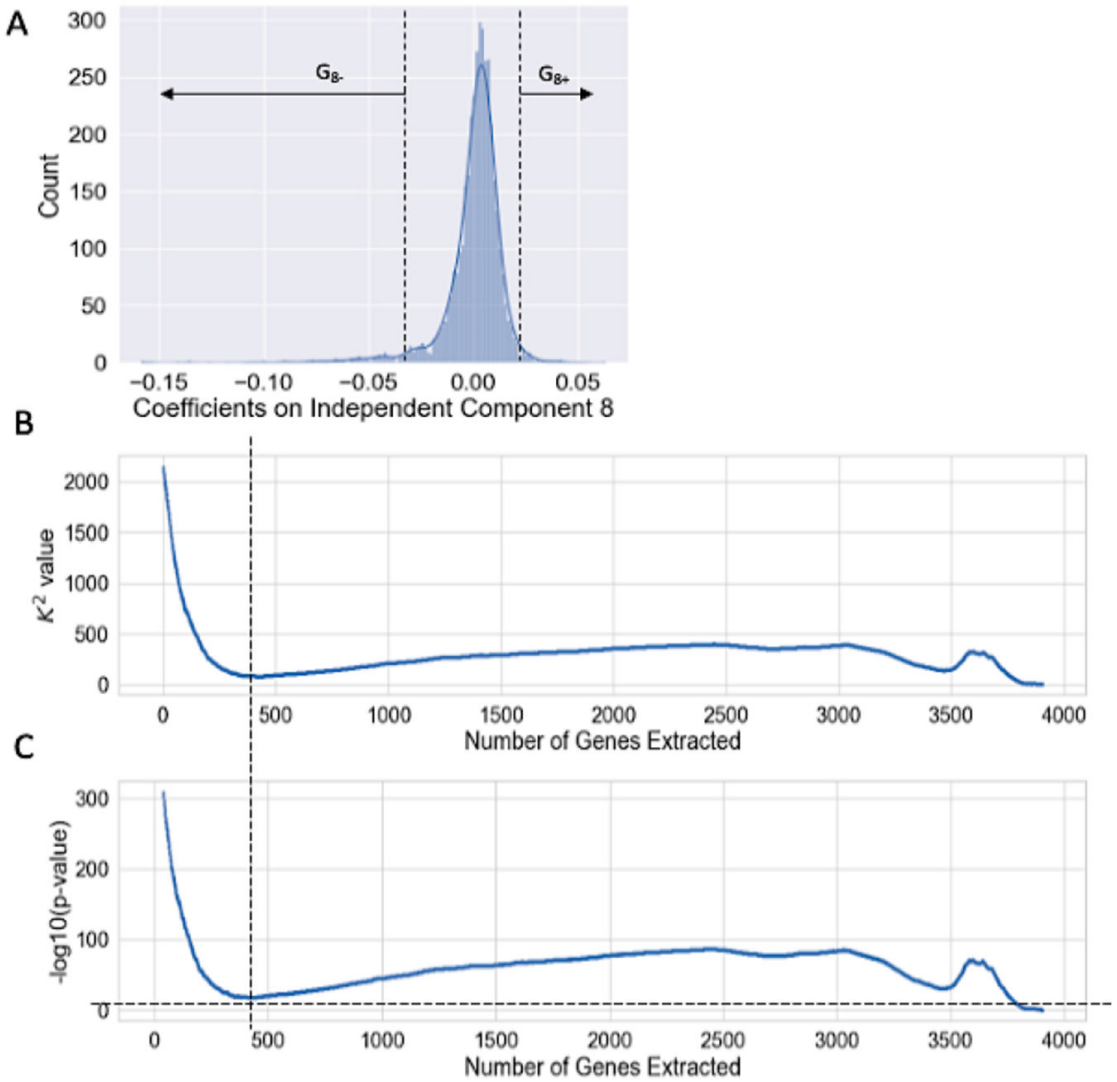
The K^2^ based procedure used to extract sets from an IC. This example is specifically to extract two gene sets G_8+_ and G_8−_ from conditionIC 8. In panels B and C, genes are sorted in order of decreasing magnitude of signal coefficient on the condition independent component.

An example of this can be seen in Figure 2, where the extraction is performed on independent component 8 of the decomposed signal matrix G of the PRECISE dataset to obtain two gene sets G_8+_ and G_8−_. Panel A shows the initial distribution of all the coefficients (per gene) in component 8 of matrix G, highly kurtotic and skewed. As we extract genes with the highest absolute coefficient the distribution becomes increasingly “normal”. Post extraction, the remaining coefficients follow the normal distribution with minimal skew and kurtosis. The vertical line in Panels B and C show the first point at which the mapping of the remaining coefficients of the component follows a normal distribution, i.e., the point at which cluster extraction is completed (p-value < 0.05, the horizontal line in Panel C). In this example, the resulting gene cluster G_8−_ has 187 genes and G_8+_ has 318.

This extraction is performed on every component $G_{i}$ of the k condition ICs to obtain at most 2k total genes sets and, on every component, $C_{j}$ of the l gene ICs to obtain at most 2l total condition sets. Elements could have associations with multiple ICs. Certain genes may not show an association to any condition IC and thus are not part of any gene set. Occasionally, coefficients on an IC are normally distributed, hence 0 clusters are extracted, or they may have a large tail in one direction only, resulting in only one cluster. However, all conditions not in a condition set by the completion of the K^2^ based extraction on all ICs, were associated with a component using the highest absolute coefficient.

## 3. EXPERIMENTAL EVALUATION

### Datasets

#### Escherichia coli:

The PRECISE dataset is a Precision RNA-seq Expression compendium for *Escherichia coli* from the Systems Biology Research group at UC San Diego [19] of 278 RNA-Seq expression profiles across 103 experimental conditions.

#### Mycobacterium tuberculosis:

A DNA microarray library of transcriptional responses to anti-TB drugs and stress conditions [30] in 63 conditions spanning 13 defined categories (grouped by mechanism of action). We combined this dataset with bedaquiline (BDQ) RNAseq data from the GEO database [31], a published set of 176 RNA-seq datasets for *M. tuberculosis* [2], and a few other transcriptomic datasets.

Additional datasets in Supplemental Materials.

### Other Details

We implement the algorithm in Python using the FastICA function in the *scikit-learn* library [32], with all the computational experiments run on Mac machine with an Apple M1 chip 3.2 GHz and 16GBs of RAM.

### 3.1 Method Comparison

The focus of Dual ICA, and most important aspect to evaluate, is the identification of interactions between gene and condition sets. However, the first step is to find gene clusters, which is done by a single ICA, and we start by evaluating the quality of these clusters.

Our findings demonstrate that ICA consistently identifies well-defined clusters that overlap with known regulons and pathways more effectively than alternative methods. This highlights the effectiveness of ICA in extracting interacting modules within the Dual ICA approach.

#### 3.1.1 Genes clusters extracted by Dual-ICA have high overlap with known regulons in E. coli

The gene sets extracted in Dual ICA were compared to 5 other clustering methods: KMeans, PCA-KMeans, Hclust, Spectral Biclustering, UMAP and WGCNA using the PRECISE dataset.

The numbers related to identified regulons reflect the enrichment of 91 that have 10 or more members of the total 275 known *E. coli* regulons. The percent overlap calculated in the last column was calculated by finding the Dual ICA gene cluster with the highest overlap for each cluster extracted from the chosen methodology. Of these best matching clusters, those that had a overlap of at least 75% were counted. To make a fair comparison of the 95 Dual ICA gene sets (from 48 components) and other unsupervised clustering methods, 95 clusters were extracted from KMeans, PCA-KMeans, Hclust, and Spectral Biclustering. PCA-KMeans reflected the output of PCA with 20 PCs (explaining 90% of variance in the PRECISE dataset) which was then clustered using KMeans clustering. Hclust was performed using the Euclidean distance, following the ward methodology. Additionally, a column clustering parameter of 48 was passed into UMAP and Spectral Biclustering function to compare condition clusters extracted from these methodologies.

For the clustering methodologies that extracted gene sets, Fisher’s Exact Test was used to find the known regulons that the clusters were enriched for. We calculated the number of total enrichments and the number of known regulons that were uniquely represented in the clusters. As seen in Table 1, Dual ICA outperforms the other methodologies. On average, the clusters extracted by Dual ICA had most total enrichments and represented the highest number of unique known regulons among the unsupervised methods. iModulons [19] exhibited similar enrichment patterns as Dual ICA gene clusters. In fact, 61 iModulons display a minimum of 75% overlap with its corresponding best matching Dual ICA gene cluster. Both methodologies initiate their procedure with an ICA on the conditions. However, the extraction process in iModulon creation incorporates pre-determined condition groupings to inform the K^2^ statistic utilized for extraction [19]. In contrast, gene clusters extracted using the Dual ICA methodology are determined independently of any condition groupings. The condition groupings are determined through a second ICA and then associated with the extracted gene clusters. This difference in extraction methodology may contribute to the higher number of total regulons represented by the iModulons.

The lowest performing methods were WGCNA and Spectral Biclustering. WGCNA extracted 18 non-overlapping gene sets ranging from size 38 to 710 and Spectral Biclustering extracted 95 gene sets also non-overlapping. Since a gene can be a member of multiple regulons, Dual ICA with overlapping genes in its extracted gene clusters was able to reflect the regulons more accurately than either of these methodologies.

The condition sets we see in UMAP are qualitatively better than those extracted in Spectral Biclustering. For example, conditions with the deletion of the *nac* gene. In UMAP, as well as Dual ICA, these conditions are in their own cluster whereas in the biclustering method, they are in separate groups with conditions that are not expected, such as those with the deletion of *crp*. Neither of these two methodologies allows for cluster overlap, thus even though UMAP encapsulates the relationships between the conditions more effectively than Spectral Biclustering, it cannot show multiple condition set relationships due to various gene set behavior that the Dual ICA methodology can.

#### 3.1.2 Dual-ICA improves condition clusters and associations over single ICA

Sastry used the PRECISE dataset [19] to demonstrate that consistent regulatory components, robust to additional data integration, can be identified in expression datasets spanning various conditions. The authors employed a semi-supervised approach to identify gene sets, called iModulons, using the S matrix obtained from running ICA on the conditions. The authors then examine the activity levels of the iModulons (strongly associated with regulons) across different conditions using the A (mixing) matrix of the ICA decomposition.

**Figure 3:**
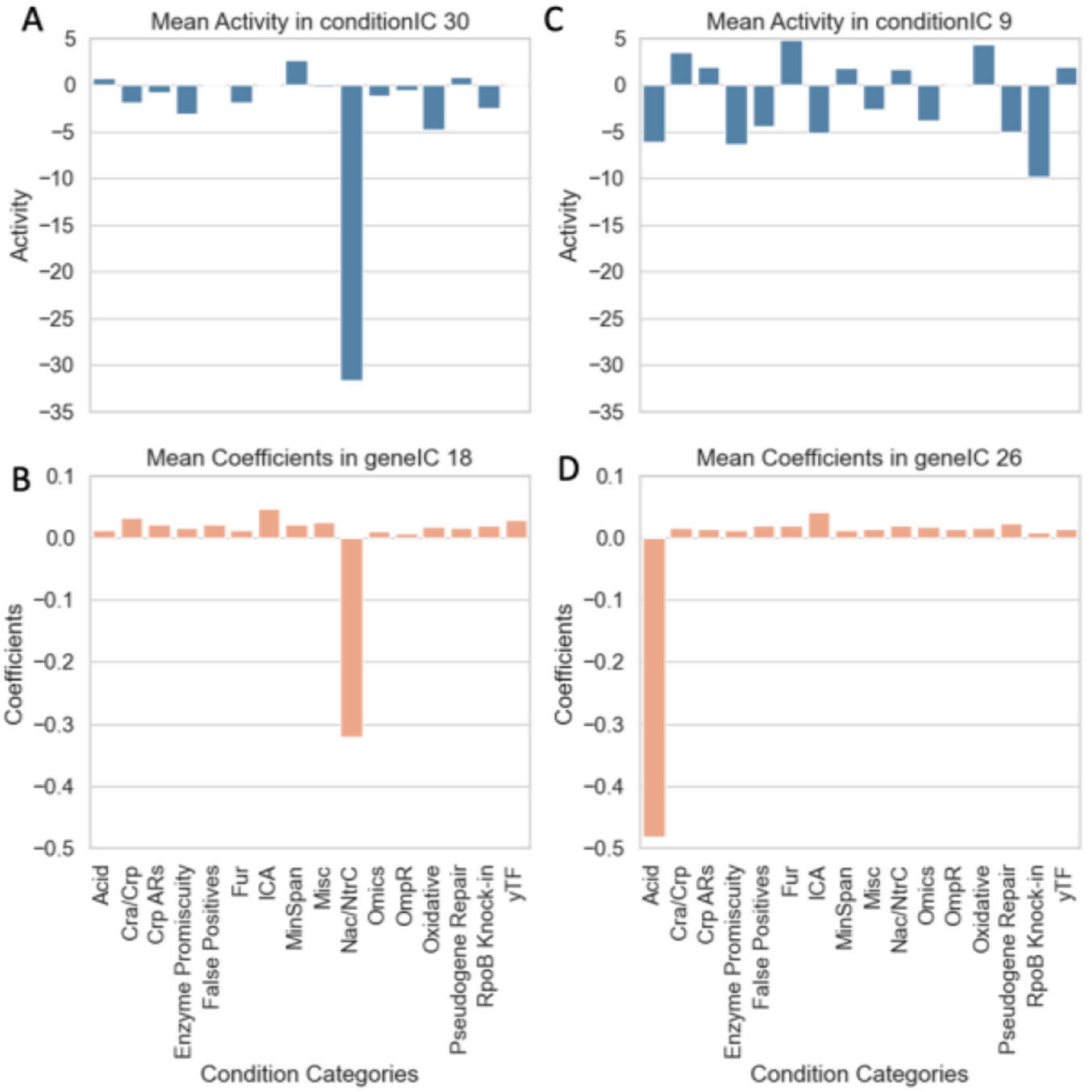
Mean Activity in select conditionICs (blue) from mixing matrix of first ICA, and mean coefficients in select geneICs in signal matrix of second ICA (orange).

In the Dual ICA framework, we observe a similar phenomenon in the mixing matrix A obtained from the ICA decomposition on conditions, represented as $M_{\left[nXm\right]}=G_{\left[nXk\right]}\;A_{\left[kXm\right]}$. For example, Sastry et al.,[19] observed increased activity of conditions when *E. coli* is grown on various nitrogen sources in the signal for the Nrp + RpoN iModulon, which encompasses regulators related to growth on nitrogen. Likewise, we see the mean activity for the nitrogen growth conditions in Figure 3A is highest in conditionIC 30, from which the gene set related to nitrogen growth is extracted from. In this case, the mean coefficients of condition categories in geneIC 18 (the IC with the strongest signals for the nitrogen growth conditions in signal matrix C, obtained from decomposition on genes) is similar in magnitude to the activities seen in Panel A.

While the mixing matrix can aid in qualitative analysis of prominent activities, such as those exhibited by the nitrogen growth conditions, it is not as effective for all types of conditions. For example, conditions related to moderate acid stress show much more diluted activity across signals in Figure 3C. Additionally, there are no conditions that show exceptional activity for the ICs the GadX, GadXW and EvgA iModulons are extracted from. These specific gene sets are known to be involved in pH homeostasis, yet there is no unique and unambiguous activity by the acid response conditions for these sets. This dilution of activity for conditions across multiple ICs, makes it insufficient to associate some categories of conditions with the genes they most affect and vice versa.

Therefore, in the Dual ICA methodology, a second ICA (on the transpose of the data matrix) is performed on the genes to uncover condition-specific signals. In the case of acid stress, we see in Figure 3D that these conditions have a clear signal on geneIC 9, which is not evident in Panel C. Furthermore, gene IC 9 strongly and uniquely associates with condition IC 9 (using coefficients of linear regression), which contains the acid-stress-related genes. When condition clusters are extracted from the geneICs of the signal matrix C and are associated with gene sets, we observe associations expected but not seen in the single ICA methodology. For instance, interacting module $C_{9+}\;x\;G_{9+}$, contains conditions related to acid stress response and the related genes.

In summary, the Dual ICA approach improves the analysis of relationships between condition sets and gene sets beyond what can be inferred from the mixing matrix, by capturing signals in both genes and conditions via the two ICAs performed, which provides greater clarity in the clustering and association of conditions.

### 3.2 Interacting Modules extracted from *E. coli* coincide with known gene - condition associations

The advantage of the Dual ICA methodology lies in its ability to take this method further and explicitly identify groups of genes that respond to specific groups of conditions, offering more functional insight. In our unsupervised analysis of the PRECISE dataset, we employed a kurtosis scree plot to identify 48 condition-specific ICs (condition ICs) and extracted approximately 95 interacting gene sets. Each IC was divided at most into two groups of genes, one with positive coefficients and one with negative coefficients (e.g. $G_{30+}$ and $G_{30-}$ are extracted from condition IC 30). As seen in Table 2 of the gene clusters extracted from the PRECISE dataset include 2527 of the 3923 total genes, ranging from 4 members to 338, averaging to about 102 members each.

**Figure 4:**
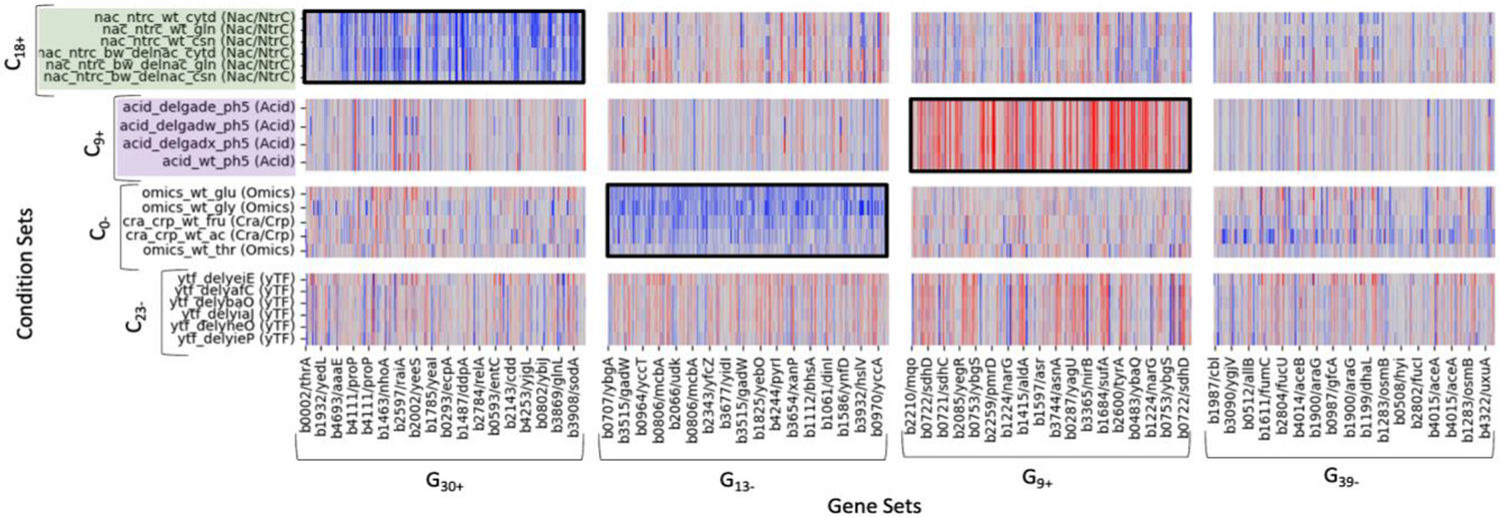
Truncated version of LFC matrix showcasing selected interacting modules. Black outlines show significant interacting modules. These typically show strong signals for up or down regulation.

Among the genes mapped, 1797 are members of multiple gene sets. On average 63.3% of a given regulon maps to its best matching gene cluster(s). 48 condition sets were extracted from 30 gene ICs (some ICs resulted only in 1 cluster and some in 0). We find that the condition sets extracted through the Dual ICA methodology map to a pre-existing classification-based type of environmental stress or pathway. Figure 4 shows a truncated version of a reordered LFC matrix (see full heatmap in Supplemental Figure 1). For each possible interacting module, we extracted the LFCs for each gene-condition pair in the module. Genes and conditions could appear in more than one module. Of the 16 gene-condition set combinations depicted in Figure 4, only 3 show statistical significance. These significant interactions exhibit consistent up or down regulation within the block.

There are total of 83 significant gene-condition IC associations (out of 48 x 30 = 1440 total tested). The interacting modules outlined in black in Figure 4 correspond to genes and conditions extracted into modules from these significantly associated gene IC and condition ICs (see Figure 1).

The significant interacting modules align with expected gene behavior in the various condition sets. For instance, gene IC 18 was found to be significantly associated with condition ICs 7, 8, 17, 30 and 37. A single cluster, $C_{18+}$ (highlighted green in Figure 4), was extracted from this geneIC. It consists of conditions that investigate regulators of nitrogen assimilation, *nac* and *ntrC*. They involve the growth of *E. coli* on various nitrogen sources including cytosine, cytidine, glutamine, and NH_4_Cl. $G_{30+}$ is one of the gene clusters extracted from the interacting condition ICs. As seen in Table 2, the cluster has a total of 189 genes extracted from the positive coefficients on condition IC 30. It encompasses 94.6% of the genes in the Nrp + RpoN iModulon, which Sastry et al., [19] showed has a higher activity in these nitrogen growth studies than other conditions. Additionally, as expected $G_{30+}$ is significantly enriched by the Fisher’s Exact Test for the *ntrC* and *nac* regulons. Additionally, the strongest signals in this module are of *glnK, amtB, rutABCDEFG, astABCDE. Glnk* and *amtB* are involved in an operon regulated by *ntrC* [33]. *astABCDE* are part of the arginine catabolic pathway, regulated by arginine and nitrogen availability [34]. The *rutABCDEFG* genes are involved in pyrimidine degradation, the expression of which is controlled by *ntrC* [35].

$G_{8-}$, another gene cluster extracted, is a larger gene set, containing 246 genes that had negative coefficients on condition IC 8. It contains 100% of the GlcC iModulon and is significantly enriched for the *glcC* regulon (100% overlap), involved in the utilization of glycolate as the sole source of carbon. Reinforcing this observation, a strain of *E. coli* with deletion of *glcB, aceB, aldA, idhA*, and *glcDEF* was showed increased growth on organic nitrogen sources but with reduced glycolate production [36]. The behaviors of the two gene clusters, $G_{30+}$ and $G_{8-}$, vary in $C_{18+}$. Consistent with expectation and known behavior, gene set $G_{30+}$ is upregulated (mean LFC=+1.78) and in contrast, $G_{8-}$ is downregulated (mean LFC=−1.68). This indicates opposite behavior of the regulons in the two different gene sets.

Another example of a significant interacting module C_9+_ x G_9+_, extracted from positive coefficients on gene IC 9 and positive coefficients on condition IC 9, respectively. The conditions in this module are those in moderate acid stress (highlighted purple in Figure 4), studying genes involved in pH homeostasis *gadEWX*. This gene cluster is significantly enriched for the *evgA* regulon and includes an especially strong signal from *ydeO*. These two genes along *gadEWX*, are known regulators of the acid resistance system and related to the direct regulation of *gadE* [37]. This module also contains strong signals for genes such as *yfdVXE*, the deletion of which decreases acid resistance [38], and *asr*, the acid shock response, the transcription of which is induced under acidic conditions [39].

Thus, this application of the Dual ICA method on PRECISE demonstrated its ability to extract gene sets in an unsupervised manner that are consistent with previously extracted semi-supervised iModulons. Both methods use ICA as a first step of extracting clusters, but Dual ICA does not need knowledge of conditions grouping to extract gene clusters as needed in the extraction of iModulons. Additionally, Dual ICA was able to quantify the association between these gene sets and condition sets that were qualitatively observed in previous analyses.

### 3.3 Applying Dual ICA to *M. tuberculosis* transcriptomic data

As seen in select interacting modules of Table 5 we use a combination of microarray and RNA Seq data libraries of *M. tuberculosis* drug exposure. Table 4, provides a summary table of the modules extracted. After running Dual ICA on this dataset, we obtain 110 interacting gene clusters (using 55 condition ICs), showing a trend for dysregulation for 83 clusters of conditions (using 43 gene ICs). Of the 2365 associations calculated between each pair of gene-conditionICs, 129 are found to be significant. The gene clusters extracted from these the significantly associated ICs showed enrichment for a total of 63 KEGG pathways using the Fisher’s Exact Test. As seen in Supplemental Table 3, gene clusters from the Dual ICA represent the greatest number of COG pathways, compared to the gene clusters extracted from other methodologies.

Condition IC 10 is significantly associated with geneIC 5 and 25. Interacting modules C_5−_ x G_10+_ and C_25−_ x G_10+_ are extracted from these associated IC pairs. The strongest signals seen in G10+, extracted from the positive mapping on conditionIC 10, are from genes including *alkA, uvrA, uvrB, uvrD1, uvrD2, ruvA, ruvB, ruvC, dnaB, dnaE2, dnaQ, dnaZX, recA, recO, recX, radA*, and *lexA*. As expected, this cluster is significantly enriched for Base excision repair, DNA repair and recombination proteins, DNA replication, and DNA replication proteins, Homologous recombination, Mismatch repair, Nucleotide excision repair and Replication and repair pathways. C_25−_ contains libraries treated with DNA Damaging agents in the Boshoff library (levofloxacin, novobiocin, ofloxacin, UV radiation) as well as an *mcr11* (sRNA) mutant.

C_0−_ is contains a set of libraries in low iron environments across a various set of studies. It also includes clofazimine, a treatment that disrupts *M. tuberculosis* respiration. This is not unexpected as iron availability (stressor being tested in the other conditions) affects the oxidative state of a cell, causing changes in respiratory pathways that include switching to NADH dehydrogenase [40]. As expected, the significantly associated gene cluster G_42+_ contains strong signals for genes including subunits of the mycobactin synthase *mbtA, mbtB, mbtC, mbtD, mbtE, mbtF, mbtG, mbtH, mbtI, mbtJ*. Mycobactin is a siderophore that is secreted and used to bind to free iron in the medium and transport it into the cells. Predictably, this interacting module shows upregulation of these genes in these low iron conditions.

Applying the Dual ICA method to the *M. tuberculosis* dataset not only demonstrates its ability to consistently identify similar conditions across different studies, including datasets with a mixture of microarray and RNASeq data, but also highlights its effectiveness in associating these conditions with known genes and KEGG pathways. Furthermore, this method shows promising applicability across species, providing valuable insights into functional relationships between genes and conditions.

## 4. DISCUSSION

Applying the Dual ICA methodology to RNASeq data enables the extraction of interacting modules, encompassing specific sets of genes and conditions. This extraction process enables us to draw inferences and hypotheses about the underlying mechanisms of action and affected pathways.

In contrast to PCA, which focuses on maximizing variance in orthogonal dimensions, a single run of ICA was able to extract gene sets from signals that maximize non-Gaussianity and enables the examination of their behavior across conditions. Sastry et al.,[19]’s use of this method significantly enhanced the quality of identified gene clusters, referred to as “iModulons”. The Dual ICA approach further improves Sastry et al., [19] ’s method by not only *agnostically* extracting similar gene sets and going beyond examination of the mixing matrix by explicitly clustering the conditions, but also establishing an automated association between the extracted gene and condition sets.

A notable feature of the Dual ICA method is its better recovery of known clusters of genes or conditions based on biological similarities. Simulations have shown the higher order moments help ICA recover signals for effectively than PCA [16]. Although some small clusters consisted of conditions grouping with their respective studies, the majority of condition sets reveal connections across studies, linking underlying mechanisms being studied. For instance, in the *M. tuberculosis* dataset, conditions from different studies related to the growth of *M. tuberculosis* in low iron conditions are clustered together.

The Dual ICA methodology can be seen as a form of biclustering [41], which encompasses various techniques ranging from search/optimization to Bayesian approaches. These methods aim to address the challenge of identifying subsets of elements that exhibit similar behavior by simultaneously clustering the rows and columns of datasets. Similarly, by performing two distinct ICA decompositions simultaneously and using the D'Agostino criterion, we identify subsets of genes and conditions that exhibit strong associations with each other within the overall data matrix. Like traditional biclustering methods, we also encounter elements that do not belong to any subsets. Through this methodology, we uncover patterns and associations between subsets of genes and conditions, providing valuable insights into relationships and functional modules within complex datasets.

The Dual ICA methodology reveals interacting modules that correspond to known groups of genes influenced by specific drug classes. For instance, in the PRECISE dataset, genes related to the *ntrC* and *nac* regulons are upregulated in libraries grown on various nitrogen sources studying these specific regulators. Conversely, genes related to *glcC* regulon, involved in utilizing glycolate as the sole carbon source, are downregulated under the same conditions. Such interactions are not apparent in modules obtained through traditional biclustering methods, which are more susceptible to noise. While the traditional bi-blustering methods yielded modules with more extreme mean log fold changes (LFCs) than the interacting modules obtained from the Dual ICA approach, they did not align as effectively with mechanisms of action or known regulons/pathways compared to Dual ICA modules.

The gene clusters extracted using the Dual ICA methodology range up to nearly 300 genes. To further explore these larger clusters, one could perform hierarchical clustering to break down large clusters like these, utilizing the coefficients on the independent components (ICs) from which the cluster was extracted, or the log fold changes (LFCs) of the genes in the cluster across all the conditions.

To broaden our investigation of gene-condition relationships, we have the potential to incorporate additional omics datasets such as metabolomic and proteomic data, as well as essentiality data from TnSeq [3].

## Supplementary Material

1

## Figures and Tables

**Table 1:** Enrichments of *E. coli* regulons/pathways (using Fisher’s Exact Test) among gene clusters identified by various methods. 95 clusters were extracted by each method. Some are enriched for more than one known regulon. Unique enrichments describe how many regulons are represented by at least one cluster.

Methodology	# Total Overlaps with Known Regulons	# Unique Regulons Represented	# Clusters with at least 75% overlap with Dual ICA Gene Sets
KMeans	172	57 / 91	51 / 95
PCA - KMeans	150	57 / 91	54 / 95
Hclust	200	64 / 91	57 / 95
Spectral BiClustering	87	33 / 91	32 / 95
WGCNA	72	50 / 91	2 / 19
iModulons [semi-supervised]	**158**	**70 / 91**	**61 / 92**
Dual ICA Gene Sets	**208**	**70 / 91**	**--**

**Table 2:** Summary of extracted modules in PRECISE dataset using the Dual ICA methodology.

Total Genes	3923	Total Conditions	103
Number of ICs used in ICA(X)	48	Number of ICs used in ICA(X^T^)	40
# Clusters Extracted	95	# Clusters Extracted	48
Cluster Sizes	338,318,…,4	Cluster Sizes	1,…,10
# Genes in Any Cluster	2527	# Conditions in Any Cluster	103
# Genes ≥ 2 clusters (Overlaps)	1797	# Conditions ≥ 2 clusters (Overlaps)	43
# Genes in NO cluster	1368	# Conditions in NO cluster	0

**Table 3:** Select significant block modules extracted through Dual ICA using the PRECISE dataset

Module	Conditions		Genes (total)	Significant Regulons
C_18+_ x G_30+_ MeanLFC = +1.78, Regression Coeff. = +83.5 Wald p-value = 0	nac_ntrc_wt_cytd (Nac/NtrC)	nac_ntrc_bw_delnac_cytd (Nac/NtrC)	*glnK, amtB, rutABCOEFG, astABCDE,*… (189)	rpoN (ES=6.095, qval=1.25E-40, overlap= 53.2%) ntrC (ES=7.828, qval=2.08E-28, overlap=90.5%) phoP (ES=3.559, qval=3.82E-06, overlap=36.7%) nac (ES=2.874, qval=0.027, overlap=40.0%)
	nac_ntrc_wt_gln (Nac/NtrC)	nac_ntrc_bw_delnac_gln (Nac/NtrC)		
	nac_ntrc_wt_csn (Nac/NtrC)	nac_ntrc_bw_delnac_csn (Nac/NtrC)		
C_18+_ x G_8−_ MeanLFC = −0.41, Regression Coeff. = +32.4 Wald p-Value = 0	nac_ntrc_wt_cytd (Nac/NtrC)	nac_ntrc_bw_delnac_cytd (Nac/NtrC)	*gatC_1, gatC_2, gatABZY, mglABC, actP, ydcH,*…(246)	glcC (ES=3.101, qval=0.0356, overlap=100.0%) crp (ES=2.265, qval=9.39E-19, overlap=22.3%) arcA (ES=2.784, qval=5.54E-11, overlap=29.0%)
	nac_ntrc_wt_gln (Nac/NtrC)	nac_ntrc_bw_delnac_gln (Nac/NtrC)		
	nac_ntrc_wt_csn (Nac/NtrC)	nac_ntrc_bw_delnac_csn (Nac/NtrC)		
C_9+_ x G_9+_ MeanLFC = −1.68, Regression Coeff. = −59.8 Wald p-value = 0	acid_wt_ph5 (Acid)	acid_delgadw_ph5 (Acid)	*yfdVXE, ydeOP, emrKY, asr,*… (92)	evgA (ES=5.364, qval=3.68E-06, overlap=70.6%)
	acid_delgadx_ph5 (Acid)	acid_delgade_ph5 (Acid)		

**Table 4:** Summary of extracted modules in the M. tuberculosis dataset using the Dual ICA methodology

Total Genes	3293	Total Conditions	265
Number of ICs used in ICA(X)	55	Number of ICs used in ICA(X^T^)	43
# Clusters Extracted	110	# Clusters Extracted	83
Cluster Sizes	244,219,…,2	Cluster Sizes	25,19,…,1
# Genes in Any Cluster	2475	# Conditions in Any Cluster	265
# Genes ≥ 2 clusters (Overlaps)	1572	# Conditions ≥ 2 clusters (Overlaps)	144
# Ganes in NO cluster	818	# Conditions in NO cluster	0

**Table 5:** Select significant block modules extracted through Dual ICA using the *M. tuberculosis* dataset

Module	Conditions		Genes (total)	Significant KEGG Pathways
C_0_ x G_42+_ MeanLFC = +1.53, Regression Coeff. = +0.54 Wald Test p-value = 0	Dipyridyl (ironLimitatlon)	IronDefident_Day1 (Fortune)	*mbtABCDEFGHIJ, PPE37,*… (120)	Biosynthesis of siderophore group nonribosomal peptides (ES=4.566 qval=0.002) Polyketide biosynthesis proteins (ES=4.219 qval=0.002) Metabolism of terpenoids and polyketides (ES=3.198. qval=0.009)
	Clofazimine (respiration)	IronDeficient_Week1 (Fortune)		
	Deferoxamine (ironLimitatlon)	1_days_low_iron (stress)		
	GSNO_CFZ (respiration)	7_days_low_iron (stress)		
	Ascididemin_Nat_prod (ironLimitation)			
C_25−_ x G_10+_ MeanLFC = +1.35, Regression Coeff. = −0.49 Wald Test p-value = 0	Levo (DNAdamaging)		*rvuABC, dnaAB, lexA, recAX, alkA,*… (177)	Base excision repair (ES 3.265 qval=0.012) DNA repair and recombination proteins (ES=6.834 qval=0.004) DNA replication (ES=3.673 qval=0.004) DNA replication proteins (ES=3.462 qval=0.005) Homologous recombination (ES=4.887 qval=1.39E-05) Mismatch repair (ES= 4.082 qval=0.001) Nucleotide excision repair (ES=3.478 qval=0.008) Protein families: genetic Information processing (ES=2.964 qval=5.32E-09) Replication and repair (ES=6.761 qval=3.89E-13) Unclassified: genetic Information processing (ES=3.235 qval=0.004)
	Novobiocin (DNAdamaging)			
	Oflox (DNAdamaging)			
	UV (DNAdamaging)			
	mcr11_mutant (mcr11)			

## CCS CONCEPTS

• **Applied computing**→ Computational genomics; *Bioinformatics;*
