# Supplementary material for: Dual ICA to extract interacting sets of genes and conditions from transcriptomic data
Source: ACM BCB. Author manuscript; Available in PMC 2023 Dec 31. (PMC10757798; doi:10.1145/3584371.3612968)

SUPPLEMENTAL MATERIAL

Supplemental Table 1 : *E. coli* conditions metadata

A Precision RNA-seq Expression compendium for Escherichia coli from the Systems Biology Research group at UC San Diego [19] of 278 RNA-Seq expression profiles across the 103 conditions, across 16 studies.

Authors of the publication calculated transcripts per million using DESeq2 v1.22.1 and log tranformed them using log2(TPM + 1). Replicates with $R^2 < 0.9$ between log-TPM were removed. The compendium was centered using WT *E. coli* MG1655 grown on minimal media (‘control__wt_glc__1’, ‘control__wt_glc__2’). The mean expressions in these two samples were subtracted and LFCs calculated.

Additional details of each sample and study can be found in publication's Supplemental Table 1 [19].

Study	Number of Conditions in Study	Study Description
yTF	14	deletion of uncharacterized transcription factors
ICA	13	growth on various nutrient supplementation
Enzyme Promiscuity	10	growth on non-native substrates
Pseudogene Repair	8	knockouts of genes required for adaptation to low iron
Crp ARs	7	deletion of cyclic-AMP receptor grown on different carbon sources
False Positives	7	KOs of thrA, serB, pstI
Nac/NtrC	7	mutants of nitrogen assimilation genes grown on different nitrogen sources
Omics	7	wt grown on different nutrients
Cra/Crp	5	deletion of Cra, transcription factor for catabolite repression
Acid	4	deletion of genes involved in survival in low pH
Fur	4	deletion of genes involved in iron acquisition
Misc	4	growth of knockouts on various metabolites
Oxidative	4	deletion of genes involved in oxidative stress
RpoB Knock-in	4	mutations in RNA polymerase
MinSpan	3	growth of knockouts on various metabolites
OmpR	2	deletion of regulator of osmotic stress grown in NaCl

Supplemental Table 2 : *M. tuberculosis* conditions metadata

This dataset consisted of the following studies, where LFCs were calculated following the procedure in that study:

1. DNA microarray library of transcriptional responses to anti-TB drugs and stress conditions [28] of 63 conditions across 13 phenotypic categories (grouped by mechanism of action).
2. Dataset obtained as LFCs from H. Boshoff [30]
3. Dataset from the GEO database [31] of RNA expression in libraries where *M. Tuberculosis* is treated with bedaquiline at different time points. DeSeq2 was used to calculate the LFCs at T30, T180 and T360 with T0 as reference, where $LFC = \log_2\left(\frac{normalized\ counts\ in\ treatment}{normalized\ counts\ in\ reference}\right)$.
4. A published set of 176 RNA-seq datasets for *M. tuberculosis* [2]. The authors cite the previous *E. coli* study in their LFC calculation [see above]
5. 9 gene knockout strains from E.J. Rubin’s lab at Harvard Medical School (not published)
6. Dataset of *M. tuberculosis* grown in stationary and non-replicating phase (NRP) [GSE100097]. DeSeq2 was used to calculate the LFCs for the stationary and NRP conditions, with exponential phase as the reference.
7. Dataset from lab of S. Fortune (Harvard Medical School) consisted of : alternate carbon sources, different levels of iron in media, RIF resistant mutants in the Beijing strain [GEO Accession : GSE67035]. DeSeq2 was used to calculate the LFCs with growth on glucose as the reference

The categories of these datasets were labeled as following (only for author viewing purposes, no effect on clusters generated). For further details on these conditions, see the publications referenced.

Category	# conditions	Category	# conditions
?	10	lipid	5
aminoimidazoles	2	Δmcr11	1
antibiotic	2	miceBMDM	5
Aromatic-Amides	3	miceNF	1
AX	4	ΔmihF	3
base	5	Δmrsl	11
biofilm	1	MTS1338	1
btz043	3	nitrohetrocyclic	1
carbonSource	2	redox	5
cellWall	4	respiration	23
ΔcnpB	1	resus	1
ΔcsoR	1	rho depletion	10
dapsone	3	Knockouts	9
ΔdarG	1	smx	3
degradosome	4	sq109	3
delam	3	starvation	1
dg_inh	3	Stiens	6
DNA-damaging	6	JSF	2
ΔvapC11	1	kinase	25
verapamil	3	levofloxacin	1
ITM_04	8	Fortune	3
dormancy	2	Δesx_1	1
dosR-associated	2	fat_cells	1
ΔeccE1	5	genotoxic	1
ethambutol	3	growth	2
ΔespL	1	hs2	1
hypoxia	22	transcription	2
inhA inhibitors	4	translation	5
Iron-Limitation	3	Transcriptional start-site profiling	3
stress	6	linezolid	3
sutezolid	3		

Supplemental Table 3 : Comparisons of methodologies using *M. tuberculosis* dataset

Comparisons of methodologies using *M. tuberculosis* data through 25 COG Pathways. KEGG pathways were not used (as in main text) because they include hierarchical pathways, which are difficult to evaluate with a single number. The input to all methodologies except WGCNA is LFC data (WGCNA used available expression data). Enrichment was calculated using the Fisher’s Exact test.

As with the *E. coli* method comparisons, we use a comparable number to Dual ICA of 110 for gene clusters and 48 condition clusters for methods that require inputs. In this case, it is clear gene clusters extracted from Dual ICA outperform those extracted from other methodologies.

Methodology	# Total overlaps with COG Pathways	# Unique COG Pathways Represented	# clusters with at least 75% overlap with Dual ICA Gene Set
KMeans	42	14 / 25	54 / 110
PCA - KMeans	33	12 / 25	51 / 110
Hclust	45	14 / 25	54 / 110
Spectral BiClustering	17	8 / 25	11 / 110
WGCNA	15	13 / 25	1 / 14
iModulons [semi-supervised]	12	8 / 25	7 / 80
Dual ICA Gene Sets	75	17 / 25	-

Supplemental Figure 1: *E. coli* LFC Heat map

Reorganized input LFC matrix to reflect the extracted interacting modules. For each possible interacting module, we extracted the LFCs for each gene-condition pair in the module. The conditions and genes are reordered based on cluster relationships. Those outlined in black are extracted interacting modules from significant associations. All the conditions can be seen here. However, not every gene is labeled.

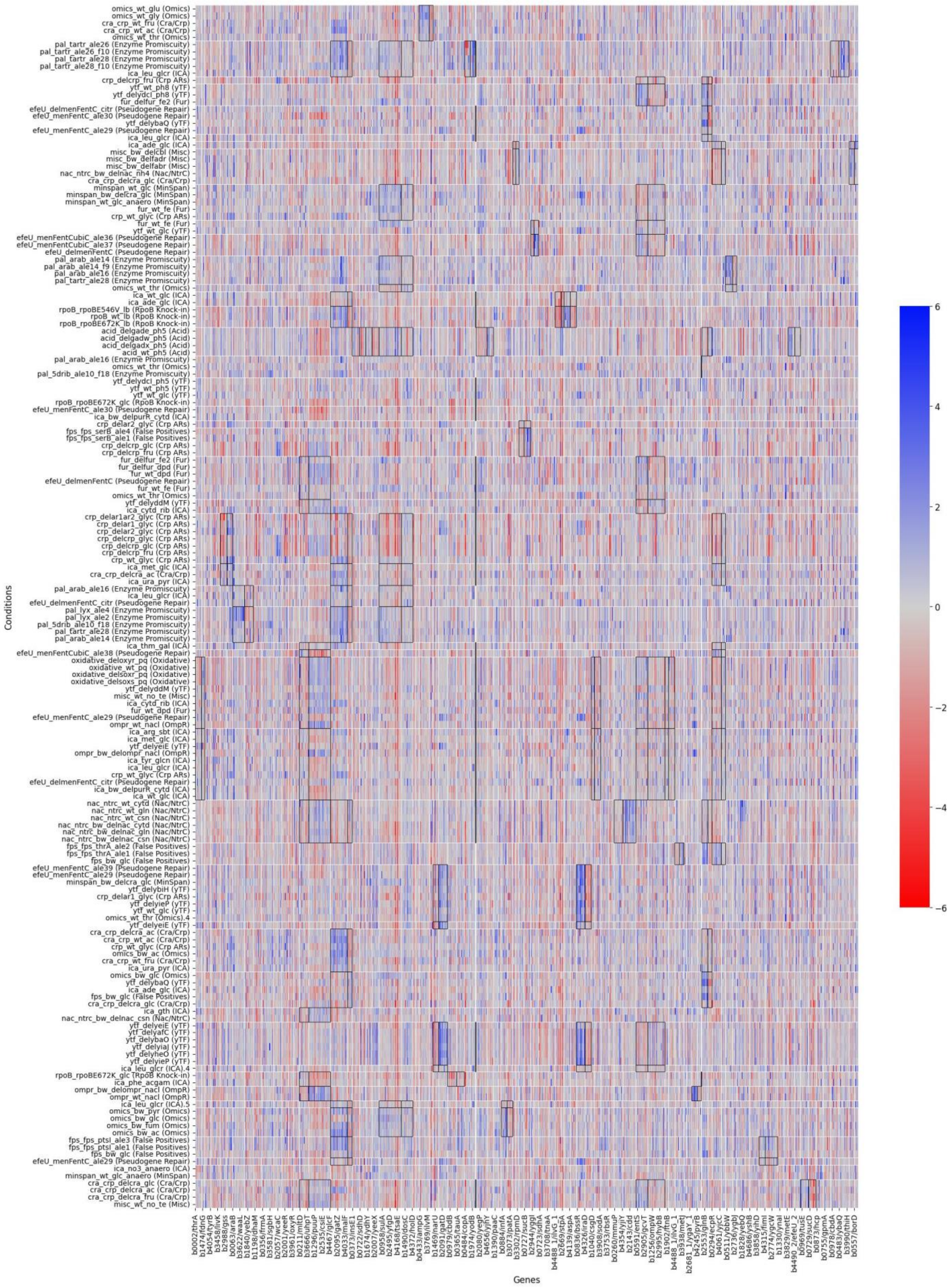

Supplemental Figure 2: ***M. tuberculosis*** **LFC Heat map**

Reorganized input LFC matrix to reflect the extracted interacting modules. Those outlined at extracted blocks from significant associations. All the conditions can be seen here. However, not every gene is labeled.

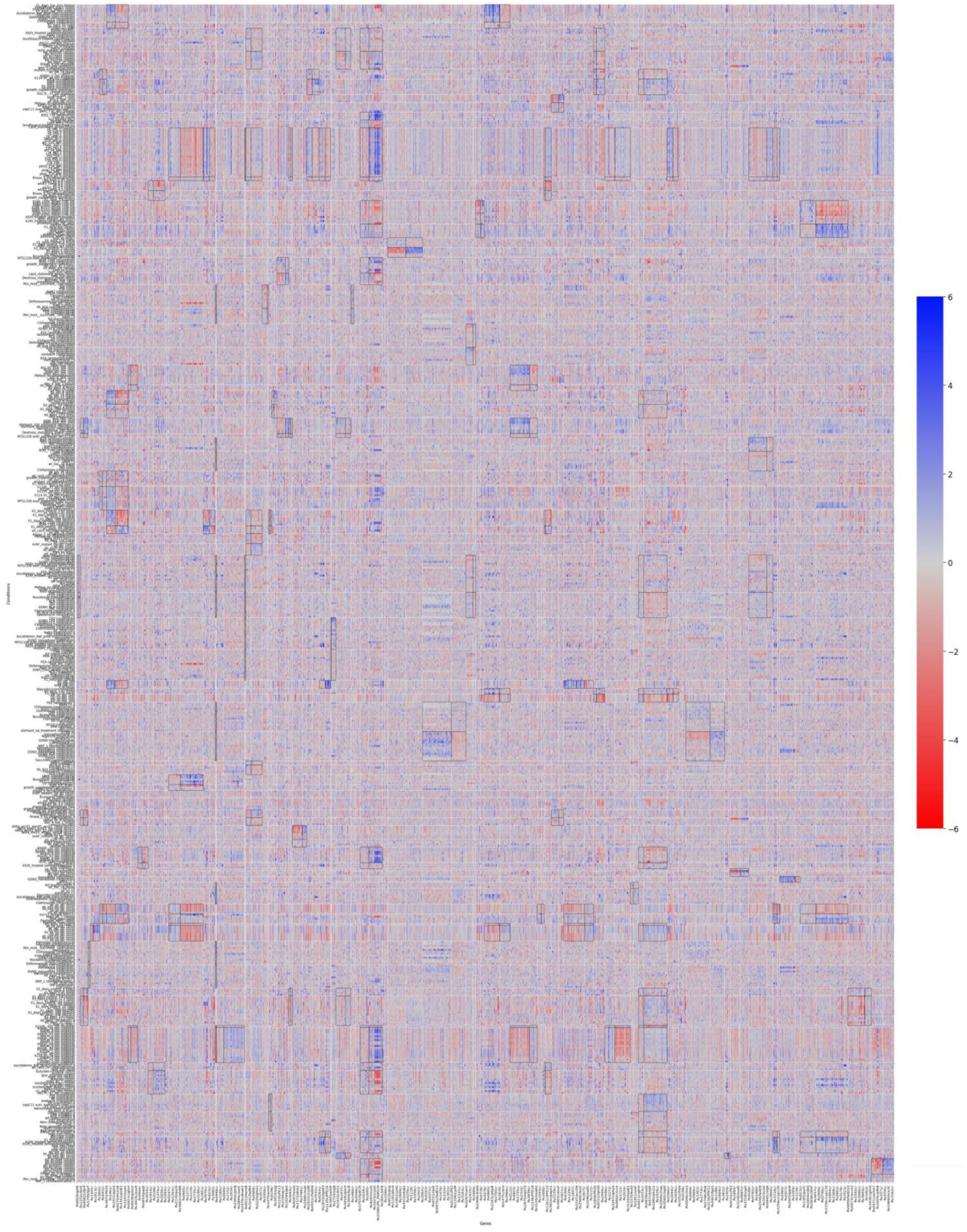

Supplement: 1 [file NIHMS1954230-supplement-1.pdf]
